# Effectiveness of combined seeds (pumpkin, sunflower, sesame, flaxseed): As adjacent therapy to treat polycystic ovary syndrome in females

**DOI:** 10.1002/fsn3.3328

**Published:** 2023-03-25

**Authors:** Naveed Rasheed, Aftab Ahmed, Farhana Nosheen, Ali Imran, Fakhar Islam, Rabia Noreen, Anamika Chauhan, Mohd Asif Shah, Yuosra Amer Ali

**Affiliations:** ^1^ Department of Home Economics Government College University Faisalabad Pakistan; ^2^ Department of Nutritional Sciences Government College University Faisalabad Pakistan; ^3^ Department of Food Science Government College University Faisalabad Pakistan; ^4^ Department of Home Science Chaman Lal Mahavidyalay Landhora Haridwar Uttarakhand India; ^5^ University Center for Research & Development Chandigarh University Gharuan, Mohali Punjab India; ^6^ Department of Food Sciences, College of Agriculture and Forestry University of Mosul Mosul Iraq

**Keywords:** body mass index, endocrine, polycystic ovarian syndrome, seed cycling, ultrasound

## Abstract

The formation and release of eggs during ovulation are impacted by high amounts of androgens. Seed cycling is powerful in the treatment of polycystic ovary syndrome (PCOS). For efficacy studies, 90 women with PCOS, between 15 and 40 years were selected from the department of gynecology, Tertiary care unit. Women with PCOS were divided into three groups (T_0_, T_1_, T_2_) (20 women/group). Among these three groups, the first was the control group (T_0_). The second group was the experimental group (T_1_). In T_1_, 20 women with PCOS were treated with a portion control diet and METFORMIN 500 mg tab/day for 90 days. The third group was also an experimental group (T_2_). In this group, 20 women with PCOS were also treated with another treatment plan for 90 days, in which portion control diet and seed cycling were included. During the 12‐week efficacy trial, the highest follicle stimulating hormone (FSH) levels were found in the control group T_0_ (8.18 ± 0.13 mIU/mL). In T_2_, FSH falls from 6.545 ± 0.16 mIU/mL to 3.51 ± 0.13 mIU/mL throughout a 12‐week period. Overall, a portion‐controlled diet and seed cycling reduced FSH levels by 1.2% to 2.5%. LH value was 10.118 ± 0.1874 IU/L in the control group (T_0_), which climbed 12.82 ± 0.15 IU/L, but decreased from 10.62 ± 0.26 IU/L to 9.79 ± 0.17 U/L and 11.015 ± 0.24 IU/L 6.217 ± 0.167 IU/L in the other groups (T_1_, T_2_). It was determined that the LH levels were reduced by 1.5%–2% in T_2_. Conclusively, the seed cycling approach is effective and has significant results in women with PCOS. Seed cycling improves hormonal disturbance in women which promotes a healthy life.

## Graphical abstract

The formation and release of eggs during ovulation are impacted by high amounts of androgens. Seed cycling is powerful in the treatment of PCOS. For efficacy studies, 90 women with PCOS, between 15 and 40 years were selected from the department of gynecology, Tertiary care unit. Women with PCOS were divided into three groups (T_0_, T_1_, T_2_) (20 women/group). Among these three groups, first was control group (T_0_). The second group was experimental group (T_1_). In T_1_, 20 women with PCOS were treated with portion control diet and METFORMIN 500 mg tab/day for 90 days. Third group was also an experimental group (T_2_). In this group 20 women with PCOS were also treated with another treatment plan for 90 days, in which portion control diet and seed cycling were included.

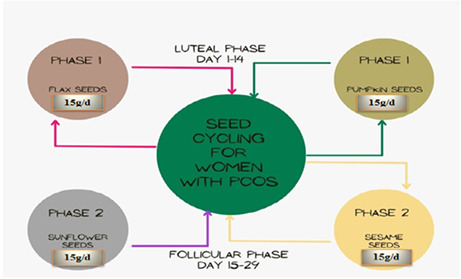

## 1 INTRODUCTION

Polycystic ovarian syndrome (PCOS) was first characterized in 1935 it is occasionally referred to as *Stein‐L eventhal* Syndrome. Between 4% and 12% of women of reproductive age are affected by this exceedingly prevalent illness (Amini et al., 2015). On ultrasonography, there are more than 10 follicles visible in the ovaries of PCOS patients. Anovulation, infertility, hirsutism, hyperandrogenism, acne, and hair thinning are some of the typical symptoms. Seventy percent of PCOS patients have functional ovarian hyperandrogenism (Nagarathna et al., 2014). The complex illness known as a PCOS causes a woman's ovaries to typically be larger than average. The term “polycystic” refers to ovaries with several cysts or follicles that infrequently mature or produce fertile eggs. Particularly in women who are infertile, PCOS is extremely common. High levels of estrogen, testosterone, and luteinizing hormone (LH), along with a reduction in the release of follicle‐stimulating hormone, are the hallmarks of PCOS (FSH). Numerous issues with the hypothalamic–pituitary‐ovarian axis as well as malignancies that produce androgen are linked to this disease (Makki et al., 2013). Growing data in recent years has suggested that PCOS therapy and control may benefit from the use of natural plant‐based solutions (Mina et al., 2020). Dietary considerations, including as anti‐inflammatory foods, may have a significant impact in reducing PCOS metabolic problems. One of the oldest crops, flaxseed has been grown since the dawn of civilization. Flaxseed is scientifically known as *Linum usitatissimum,* in latin which means “extremely beneficial” (Laux et al., 2011). Because of its high concentrations of lignans, fiber, and α‐linolenic acid (ALA, an omega‐3 fatty acid), flaxseed is becoming a crucial functional food element (Islam et al., 2023; Rizvi et al., 2021). Reduced risk of cardiovascular disease, atherosclerosis, diabetes, cancer, arthritis, and osteoporosis, autoimmune and neurological disorders are only a few potential health benefits of flaxseed oil, fibers and lignans (Rizvi et al., 2021). Flax protein supports the immune system and aids in the treatment and prevention of heart disease (Sepidarkish et al., 2019). Pumpkin seeds are very nutrient‐dense and abundant in nutraceuticals, such as unsaturated fatty acids including palmitic, stearic, oleic, and linoleic acids (Stevenson & Jarillo, 2007). These ω‐6 and ω‐3 essential fatty acids have great nutritional effects and are crucial components of numerous metabolic processes (Miura, 2013). Increases in uterine weight, mammary gland, bone density, and prevention of hyperlipidemia, the sign of estrogen‐like activities in ovariectomized females, have been documented following phytoestrogen therapy with pumpkin seeds extract. RATS, Sprague–Dawley (Gossell‐Williams et al., 2008). Pumpkin seeds also include the beneficial ω‐3 fatty acids that can help control the elevated insulin and cholesterol levels associated with PCOS. They also include beta‐sitosterol, which can reduce too much androgen and treat PCOS symptoms like hirsutism, acne, and weight gain (Reddy et al., 2016). A major oilseed crop farmed around the world is the sunflower seed (*Helianthus annuus* L.; Stefansson, 2009). A little sunflower seed is a potent source of fiber, protein, healthy unsaturated fats, vitamin E, selenium, copper, zinc, folate, iron, and phytochemicals, among other essential elements. With a global production of over 10.6 million metric tons in 2006, sunflower oil placed fourth in terms of production behind palm, soy, and rapeseed oil (Stat, 2008). One of the oldest oil seed crops, sesame (*Sesamum indicum* L.) also called as “*Konjed*” or “*Samsam”*) grows extensively in tropical and subtropical regions and is a staple of the Middle Eastern diet (Bedigian, 2004). This seed is a significant source of the active components in antiseptics, bactericides, vermicides, disinfectants, and anti‐tubercular agents. It is also a significant source of oil (44%–58%), protein (18%–25%), and (13.5%) carbohydrates (Fukuda et al., 1986). Sesame has been touted in traditional medicine as a helpful treatment for oligo‐menorrhea, foetal abortion, boosting sexual inclination, and sperm production (Yavari et al., 2016). To our knowledge, however, no scientific study has yet examined its impact on menstruation (Kamali & Shokri, 2012). This study was design to explore possible nutritional health benefits among the female patients of polycystic ovary syndrome in reproductive age. The hormonal levels LH and follicle stimulating hormone (FSH) of such females fluctuate respectively. The evaluation of change in these hormones was done through biochemical analysis of patients with disturbed hormonal levels.

## 2 MATERIALS AND METHODS

All the raw material, processing into final product, adoptive protocols and adoptive measurements are duly approved and according to the guidelines of institutional ethic and research review committee with study no ERC 5214.

### 2.1 Procurement of raw materials

Seeds (Flaxseed (*Linum usitatisimum*), sesame seeds, sunflower seeds, and pumpkin seeds) were obtained from the Ayub Agriculture Research Center, then grounded and packed into plastic zip bags in compliances with institutional approval ERC 5214.

#### 2.1.1 Preparation of seeds

Seeds were taken from Ayub agriculture research center. Grinded into powdered form through an electrical grinder then 15 g of each seed was weighed by electrical weight balance. This weighed quantity was later on packed into plastic zip bags.

### 2.2 Experimental design

Ninety women with PCOS, between 15 and 40 years were selected from the department of gynecology, Tertiary care unit from central hospital Sheikhupura Pakistan.

### 2.3 Study design and subjects

**FIGURE 1:**
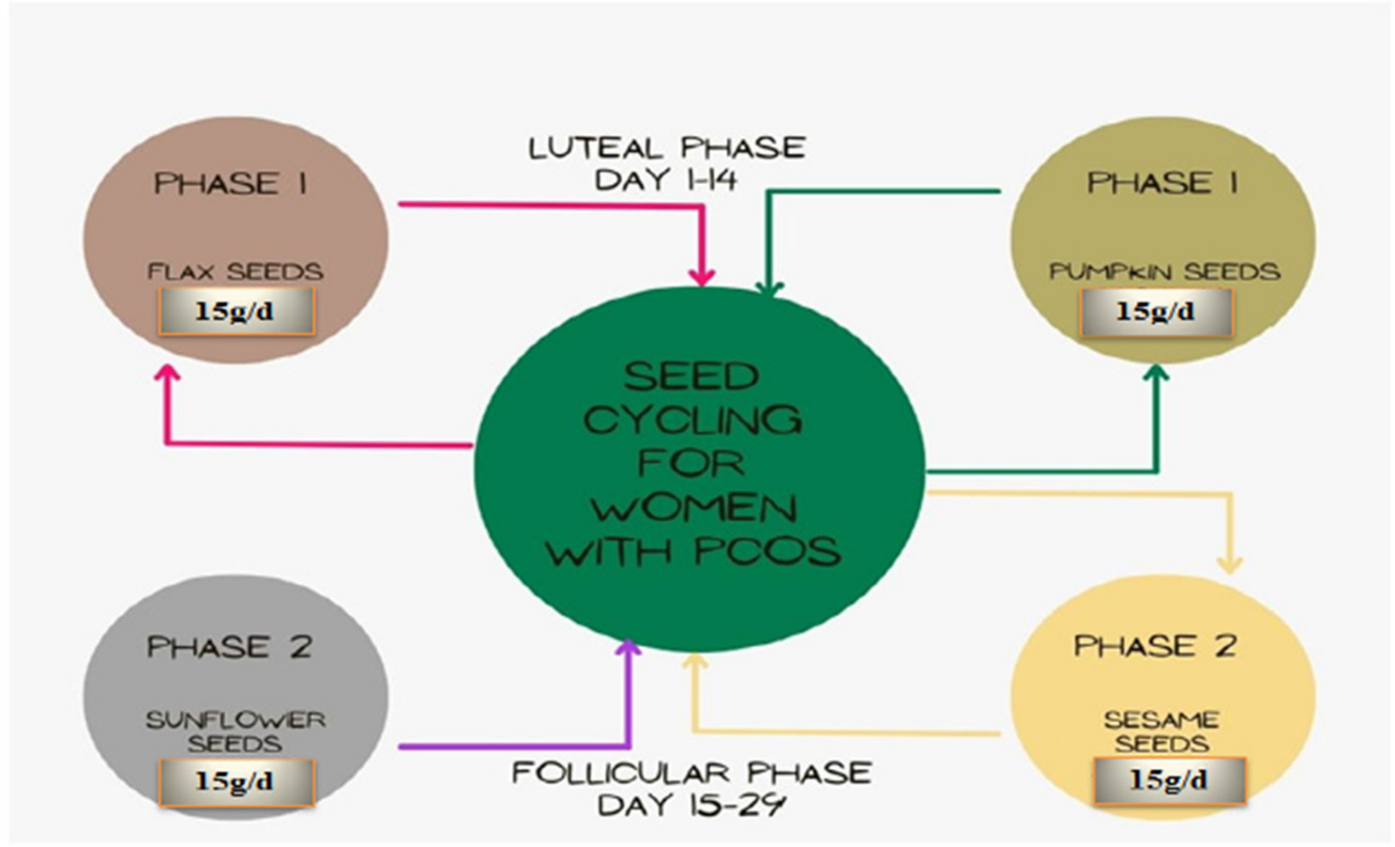
Seed cycling for women with polycystic ovary syndrome.

The study was approved by the Institutional Animal Care and Ethics Committee (ERC 5214). Moreover, it is submitted that the likelihood and degree of discomfort to human's/animal subjects expected during this study was not greater than those usually faced in daily life or during the performance of routine physical examinations or tests. Furthermore, no genetically modified organism is being used in this study and during this study there was neither any hazard to the environment nor any chance of COVID‐19 dissemination. Likewise, we followed the tenets of declaration of Helsinki and Stockholm Convention. Moreover, the human subjects were enrolled after taking their volunteer consent after specific inclusion and exclusion criteria. Furthermore, before the onset of research all the subjects were again demonstrated and asked for their final willingness. Women with PCOS were divided into three groups (T_0_, T_1_, T_2_) (20 women/group). All groups were treated with different treatment plans. Among these three groups, first was control group denoted with T_0_. The second group was experimental group denoted with T_1_. In T_1_, 20 women with PCOS were treated with portion control diet and METFORMIN 500 mg tablet per day for 90 days. Third group was also an experimental group denoted with T_2_. In this group 20 women with PCOS were also treated with another treatment plan for 90 days, in which portion control diet and seed cycling were included. Treatment (1): T_0_ control, Treatment (2): Experimental T_1_; tablet metformin 500 mg, Treatment (3): Experimental T_2_; 30 g seeds of flax and pumpkin each were given for first 14 days after menstruation and 30 g seeds of sunflower and sesame seeds of each were given in next 14 days after menstruation in two steps shown in Figure 1.

The used dosage was an average dosage based on the previous studies (Farzana et al., 2015; Hanem et al., 2019). Blood samples were taken from cases of polycystic ovaries before the beginning of the trial and at the end of the trial. The blood samples were separated and transferred to a serum for the necessary analysis. After completion of trail period Serum were collected for biochemical analysis again, to determine the effect of seed cycling on women with PCOS.

### 2.4 Analysis for PCOS

Ovulation evaluation may be broken down into types:

#### 2.4.1 Hormones assay test

Luteinizing hormone, FSH, thyroid stimulating hormone (TSH), and prolactin hormone tests were each resolved according to their own conventions. In order for fluctuations to represent intra‐ rather than inter‐assay variance, serum was kept at 20°C and each cycle or set of cycles was analyzed as a unit. Using a conventional double‐antibody technique and reagents, the serums were analyzed by radioimmunoassay (Odell et al., 1967; Sbihi et al., 2021).

##### Procedure for hormonal assay test

Process of blood sample collection: A band were placed 3–4 inches above the collection point around the arm (superficial vein that lies within the elbow pit) the skin is stretched tight by removing the needle cap and holding it parallel to the vein. By slowly pulling out the syringe's plunger, a 5 cc blood sample was taken. Then the wrap band was undone, gauze was spread on the location of the collection and the needle was taken out. The appropriate preservative, clot activator, or anticoagulant was added to the blood container into which the blood was immediately transferred. For safe and hygienic disposal, the syringe and needle were placed in the proper “sharp container” (Haddad et al., 2019; Sherman et al., 1976).

##### Normal levels of hormonal tests

These biochemical analyses were performed in biochemical Analysis Laboratory of Central City Hospital & Infertility center, Sheikhupura (Table 1).

**TABLE 1:** Biochemical analysis of hormones.

Sr. no.	Test type	Gender	Age	Normal values
1.	TSH	Female	Adults	0.5–5.0 mIU/L
2.	LH	Female	Adults	1.09–9.2 IU/L
3.	FSH	Female	Adults	1.5–12.4 mIU/mL
4.	Prolactin	Female	Adults	<25 ng/mL

#### 2.4.2 Dietary studies

Diet history including food habits was taken during an interview with patients including food likes and dislikes.

#### 2.4.3 Nutrients intake

Two types of menus were designed according to patients BMI. Daily energy and nutrients intake (e.g., protein, fat, vitamins, minerals were calculated for all patients using food intake analysis system FIAS).

##### Diet plan

**FIGURE 2:**
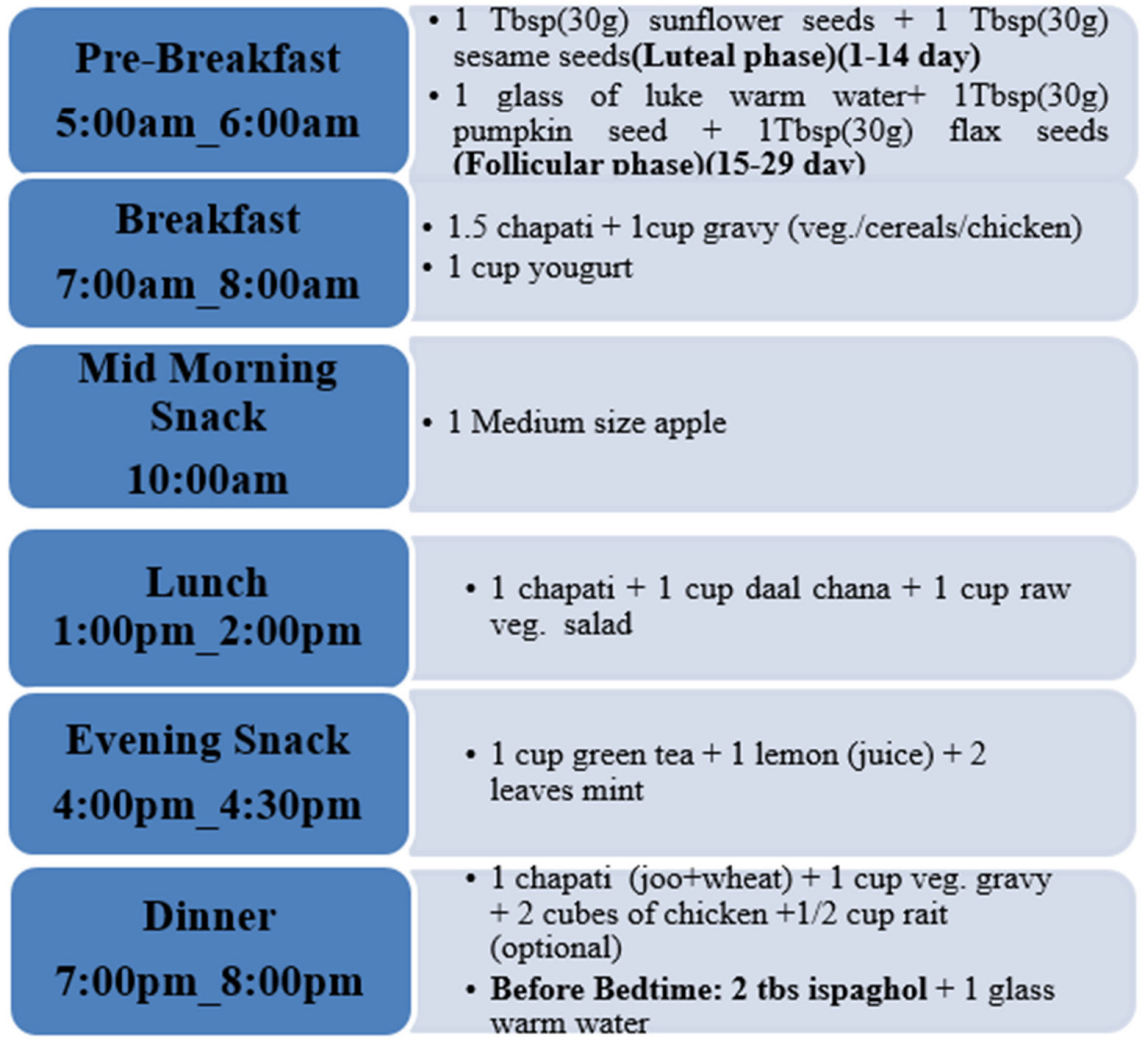
Flow sheet depicts the diet plan.

A healthy diet for patients with PCOS included 1500 kcal, three meals per day (breakfast, lunch, and supper), and two snacks. This diet was composed of 40% carbohydrates, 20% protein and 40% protein (mid‐morning, evening; Figure 2).

###### Instructions

1. Brisk walk for 40 min
2. Drink 10–12 glasses of water daily
3. Avoid all junk items, fatty meals, preserved meals and bakery items
4. 60 min regular walk and 10 min deep breathing in early morning

#### 2.4.4 Body measurements

##### Anthropometric measurements

Measurements of height, weight, and body mass index (Mitch & Klahr, 1993).

##### Height

Height was measured to the nearest 0.5 cm with subject standing with head, shoulder but tocks and heals vertically aligned and bare footed.

##### Weight

Weight was measured to the nearest 0.1 kg with light clothing and without shoes. Body weights of all participants were recorded at the base line after 6 and 12 weeks of the following regimen.

##### Body mass index

Body mass index was calculated according to Mitch and Klahr (1993), as the weight (kg)/height (m^2^). The prevalence of overweight or obesity was determined.

##### Menstrual cycle history

It included age at menarche, frequency, duration and severity of menstrual flow and history of dysmenorrheal. In addition, information on history of difficulty in conception was collected (Jayaprakasan et al., 2007).

### 2.5 Statistical analysis

The attained data were statistically studied using SPSS‐PC statistical package software and the type of test is ANOVA followed by Duncale at .05 significant (SAS, 2004).

## 3 RESULTS AND DISCUSSION

### 3.1 Efficacy studies

The aim of study was to check the effect of seed cycling on women with PCOS along with portion control diet. For efficacy studies, 90 women with PCOS, between 15 and 40 years were selected from the department of gynecology, Tertiary care unit. Women with PCOS were divided into three groups (T_0_, T_1_ and T_2_) (20 women in each group). All groups were treated with different treatment plans. Among these three groups, first was control group denoted with T_0_. The second group was experimental group denoted with T_1_. In T_1_, 20 women with PCOS were treated with portion control diet and METFORMIN 500 mg tablet per day for 90 days. Third group was also an experimental group denoted with T_2_. In this group 20 women with PCOS were also treated with another treatment plan for 90 days, in which portion control diet and seed cycling were included. Then blood sample was taken from patients and tested for the effect of seed cycling on women with PCOS. Afterwards, seed cycling was start for 90 days to assess its health curing potential against life style related disorders like polycystic ovary syndrome. Body weight was measured on weekly basis. The collected blood samples were analyzed for prolactin, FSH, LH, and TSH level. For better understanding, results relating to each parameter in relevant study were collectively interpreted.

#### 3.1.1 Body weight and ultrasound (A diagnostic tool)

Mean squares for body weights of women in different groups have revealed highly significant effect of treatments and study days (Table 2). It is evident from Table 2 that body weight was significantly higher in treatments (T_0_) in contrast to treatment (T_1_ and T_2_). Body weight in control group increased with passage of time ranging from 82.950 ± 2.4310 kg to 89.50 ± 0.0213 kg but in other groups increasing rate in body weight did not coincide to that of control group. Seed cycling and portion control diet given to the women, participated significantly in decreasing weight in T_1_ and T_2_ from 95.85 ± 1.0320 kg to 87.95 ± 0.62 kg and 93.10 ± 2.01 kg to 81.65 ± 0.73 kg, respectively.

**TABLE 2:** Comparisons test of weight (kg) for A*B.

Treatment	Day 0	Day 90	Mean
T_0_	82.950 ± 2.431	89.500 ± 0.02	86.225 ± 2.003
T_1_	95.850 ± 1.032	87.950 ± 0.62	91.900 ± 1.93
T_2_	93.100 ± 2.012	81.650 ± 0.73	87.375 ± 2.83
Mean	90.633 ± 0.032	86.367 ± 1.82	

Kazemi et al. (2021) discovered a reduction in ovarian cysts in PCOS women after seed cycling and portion control diet administration; nearly half of them had less cysts, while 36% had complete cyst degeneration. Seed cycling has been shown to be effective against obesity (Kazemi et al., 2021). However, our findings contradicted the findings of prior investigations in PCOS women.

#### 3.1.2 Hormonal perspectives

##### Follicle stimulating hormone (mIU/mL)

Mean squares for effect of seed cycling (Table 3) on FSH have shown significant effect FSH of Women in all groups changed significantly during 12 weeks of efficacy trial. In study, maximum FSH was recorded in T_0_ (8.18 ± 0.13 mIU/mL) respectively. During 12 weeks' trial, FSH decreased from 6.54 ± 0.16 mIU/mL to 3.5100 ± 0.1303 mIU/mL in T_2_. Overall portion control diet and seed cycling have decreased FSH level by 1.2% to 2.5%, respectively.

**TABLE 3:** Comparisons test of FSH (mIU/mL) for A*B.

Treatment	Day 0	Day 90	Mean
T_0_	5.4250 ± 0.15	8.1800 ± 0.13	6.8025 ± 0.183
T_1_	6.3750 ± 0.16	5.4550 ± 0.12	5.9150 ± 0.15
T_2_	6.5450 ± 0.16	3.5100 ± 0.13	5.0275 ± 0.13
Mean	6.1150 ± 0.14	5.7150 ± 0.17	

Polycystic ovary syndrome resulted in an increase in hormone levels, which was consistent with (Irfan et al., 2021; Singh et al., 2020). FSH levels were elevated as a result of PCOS. Several investigations have found that PCOS is connected with abnormal hormonal levels. Previously demonstrated that seed cycling has a considerable favorable influence on FSH. During the development and progression of PCOS, excessively high (T) testosterone, inhibiting estrogen (E2) secretion decreases, SHBG sex hormone‐binding globulin, and FSH production (Liu et al., 2015). Furthermore, under the synergistic impact of LH, decreased FSH causes the arrest of ovarian folliculogenesis and corpora lutea (Franks et al., 2008). In this investigation, abnormally raised plasma T and LH/FSH ratios, as well as lower levels of plasma FSH, E2, SHBG, and Progesterone in PCOS, were capable of improving sex steroid hormone balance (Somboonporn & Davis, 2004). Our findings suggest that a portion‐controlled diet and seed cycling have a positive effect on androgens in PCOS.

##### Luteinizing hormone (IU/L)

It is clear from mean squares (Table 4) that seed cycling has significantly affected LH in all studies except in women with PCOS in control group. Means for efficacy study indicated that LH value was 10.118 ± 0.1874 IU/L in control group (T_0_) that increased 12.82 ± 0.15 IU/L, while in other groups (T_1_, T_2_) reduction was from 10.62 ± 0.26 IU/L to 9.79 ± 0.17 IU/L and 11.015 ± 0.24 IU/L 6.217 ± 0.16 IU/L, respectively. It was concluded that LH value decreased 1.5%–2% in T_2_, respectively.

**TABLE 4:** Comparisons test of LH (IU/L) for A*B.

Treatment	Day 0	Day 90	Mean
T_0_	10.118 ± 0.18	12.820 ± 0.15	11.469 ± 0.26
T_1_	10.624 ± 0.26	9.795 ± 0.17	10.209 ± 0.19
T_2_	11.015 ± 0.24	6.217 ± 0.16	8.616 ± 0.16
Mean	10.585 ± 0.18	9.611 ± 0.238	

Shahid et al. (2022) found a significant improvement in LH levels in PCOS women after seed cycling. Studies identified a decrease in LH after seed cycling in PCOS women, which is consistent with our findings. According to the current study, the combination of pumpkin seeds, flaxseed, sesame seeds, and sunflower seeds has the greatest lowering effect on LH among the treatment groups. Furthermore, our findings were similar with several other studies that have shown a decrease in LH levels by seed cycling.

#### 3.1.3 Thyroid stimulating hormone (μIU/mL)

Mean squares for effect of seed cycling (Table 5) on T_2_ TSH have shown significant effect. FSH of Women in all groups changed significantly. Mean values for TSH (Table 5) for control, T_1_ and T_2_ groups were 3.22 ± 0.08 μIU/mL, 3.26 ± 0.16 μIU/mL and 2.97 ± 0.18 μIU/mL, correspondingly. It was observed that TSH in control increased from 3.22 ± 0.08 μIU/mL to 3.67 ± 0.34 μIU/mL but in T_1_ and T_2_ groups decreased from 3.26 ± 0.1642 μIU/mL to 3.07 ± 0.037 μIU/mL, and 2.97 ± 0.18 μIU/mL to 2.88 ± 0.003 μIU/mL, respectively. Conclusively, portion control diet and seed cycling has 0.5%–1% significantly reduced TSH level.

**TABLE 5:** Comparisons test of TSH (μIU/mL) for A*B.

Treatment	Day 0	Day 90	Mean
T_0_	3.22 ± 0.08	3.67 ± 0.34	3.40 ± 0.02
T_1_	3.2650 ± 0.16	3.0750 ± 0.037	3.17 ± 0.014
T_2_	2.97 ± 0.18	2.8850 ± 0.003	2.9275 ± 0.19
Mean	3.1517 ± 0.17	2.9783 ± 0.329	

TSH levels were shown to be reduced after seed cycling, according to Ullah (2022). Studies discovered a considerably higher level of FSH, LH and progesterone in PCOS subjects compared to controls. Ajith (2022) discovered elevated LH, FSH, and TSH in PCOS women. TSH levels increased, while LH and FSH levels fell, following therapy with seed cycling and a portion control diet (Irfan et al., 2021). In our study level of TSH decreased tremendously in the PCOS women treated with a combination of these seeds (flaxseeds, pumpkin, sunflower, sesame) and portion control diet.

#### 3.1.4 Prolactin

Mean square (Table 6) for effect of seed cycling and portion control diet has significantly changed prolactin level. Mean values for prolactin level (Table 6) for T_0_, T_1_ and T_2_ groups were 86.225 ± 2.0032 ng/mL, 91.900 ± 1.9362 ng/mL and 87.375 ± 2.8320 ng/mL, respectively. It was observed that prolactin level in control group (T_0_) increased from 82.950 ± 2.4310 ng/mL to 89.500 ± 0.0213 ng/mL but in T_1_ and T_2_ groups decreased from 95.85 ± 1.0320 ng/mL to 87.95 ± 0.6203 ng/mL, and 93.10 ± 2.0124 ng/mL to 81.65 ± 0.7321 ng/mL, respectively. Conclusively, portion control diet and seed cycling has 1.5%–2% significantly reduced prolactin level. According to Azin and Khazali (2022) a small increase in prolactin levels can be found in 30% of PCOS cases throughout both the follicular and luteal phases. According to Alaee et al. (2023) and Zhang et al. (2019) an increase in prolactin levels can reduce ovarian follicle size and ovulation.

**TABLE 6:** Comparisons test of prolactin ng/mL for A*B.

Treatment	Day 0	Day 90	Mean
T_0_	82.95 ± 2.4310	89.500 ± 0.02	86.225 ± 2.003
T_1_	95.850 ± 1.03	87.950 ± 0.62	91.900 ± 1.93
T_2_	93.100 ± 2.012	81.650 ± 0.73	87.375 ± 2.83
Mean	90.633 ± 0.03	86.367 ± 1.82	

### 3.2 Ultrasound (A diagnostic tool)

**FIGURE 3:**
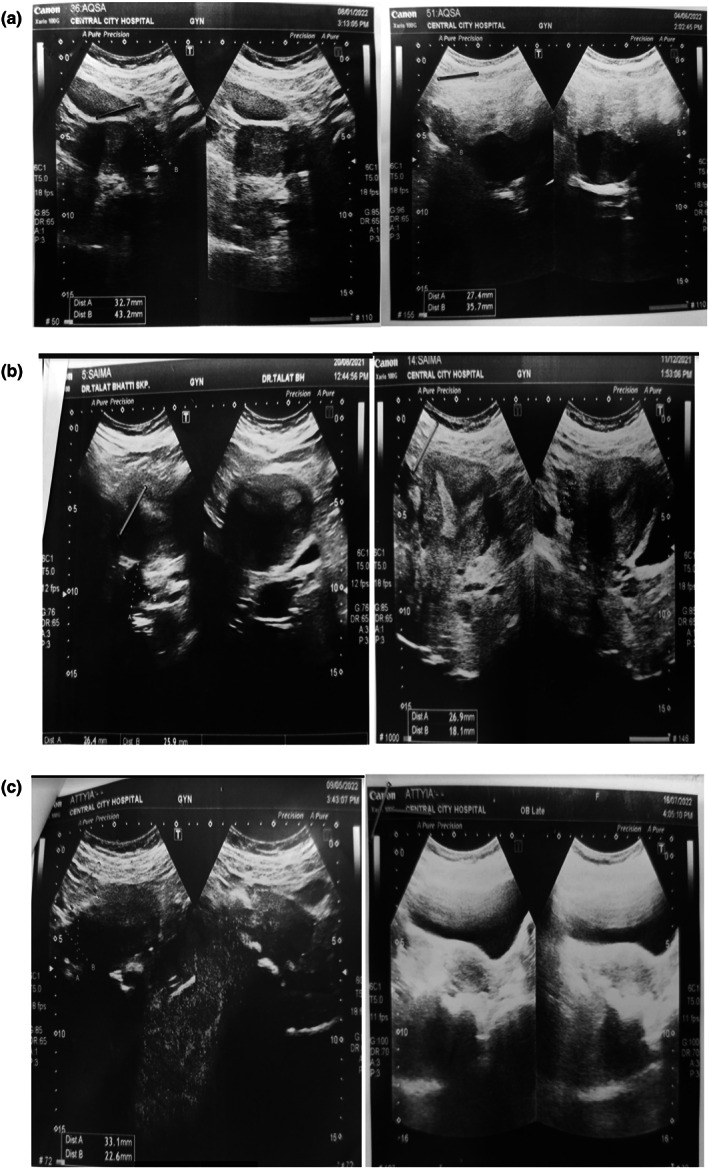
(a)Patient 1 with polycystic ovary (before and after seed cycling). (b) Patient 2 with polycystic ovary (before and after seed cycling). (c) Patient 3 with polycystic ovary (before and after seed cycling).

Woman with PCOS have bulky ovaries which can be detected and seen in a pelvic ultrasound. In a pelvic scan of a woman, suffering from these condition ovaries normally shown with normal size but cyst in ovaries seen clearly. These cysts make the presentation of ovaries bulky as an accumulated mass. After the treatment the pelvic scan of the patient was improved and presented as a clear with no cyst in the ovaries. This time the size of ovaries was normal as 2.7 × 3.3 cm with normal size, shape and volume (Figure 3a–c). When compared to other groups (T_1_, T_2_), the number of cystic follicles in the PCOS control group increased. The average number of primary and preantral follicles in the PCOS group T_2_ was less than in the other groups. In comparison to other groups (T_0_, T_1_), the number of antral follicles in the PCOS T_2_ group dropped.

Women with PCOS had a considerable increase in ovarian numbers and size, according to the findings. Khanage (2019) concluded that the increase in ovarian cyst due to the production of cystic follicles in PCOS women's ovaries. PCOS women were treated with seed cycling (flaxseed, pumpkin, sunflower and sesame seeds) improved and significantly reduced ovarian cysts (Irfan et al., 2021).

#### Hirsutism

Pumpkin seeds also include the beneficial ω‐3 fatty acids that can help control the elevated insulin and cholesterol levels associated with PCOS. They also include beta‐sitosterol, which can reduce too much androgen and treat PCOS symptoms like hirsutism.

#### Recommendations

Polycystic ovarian syndrome is common and an inevitable condition among the women of reproductive age due the sedentary lifestyle, poor dietary habits as well as high caloric intake. This condition leads to hormonal imbalance, amenorrhea, infertility and disturbance in menstrual cycle. Seed cycling approach with a dosage of 15 g of each seed in the form of two sets (pumpkin, flax) and (sesame and sunflower) for follicular and luteal phase respectively is found and effective in this condition. This approach can be utilized as snack form or may be supplemented into flour which can be consumed in daily basis. This study proves the changing in LH, FSH, TSH and S. prolactin hormonal levels which relates the condition of PCOS. The possible utilization can drop the high prevalence rate of this condition among females.

## 4 CONCLUSION

Polycystic ovarian syndrome is now becoming worst with high prevalence rate among female of reproductive age in Pakistan. Sedentary lifestyle is a key factor and high intake of calories is promoting is day by day. Seed cycling approach is a traditional approach to maintain hormonal balance in females of reproductive age. The set of seeds (flax, pumpkin, Sunflower, sesame) have antioxidants, omega 3 & 6 fatty acids, protein, carbohydrates, fiber, zinc, potassium, phosphorus, and magnesium and high levels of other trace minerals (calcium, sodium, manganese, iron, zinc, and copper) that promotes normal hormonal levels of progesterone in females. The accuracy of progesterone levels in females; also correct the fluctuated value of LH, FSH, which indicates the condition of PCOS. Seed cycling also improve TSH, prolactin level in blood that correlate the weight gain among females with PCOS. The study provides positive changes regarding the hormonal issue in females and significant results describe the capability and efficacy of the study for this condition. Therefore, by using the approach of seed cycling we can handle the highly prevailing condition of PCOS among the females of reproductive age. Further studies have to be done to explore the effect of seed cycling on PCOS for its clinical application.

## AUTHOR CONTRIBUTIONS

Conceptualization, Writing—original draft: Naveed Rasheed, Fakhar Islam, Mohd Asif Shah. Supervision, Writing—original draft and review and editing: Aftab Ahmed, Farhana Nosheen. Validation, Methodology: Ali Imran, Rabia Noreen. Formal Analysis, Investigation, Resources: Anamika Chauhan. Software: Adil Rasool. All authors have read and agreed to the published version of the manuscript.

## FUNDING INFORMATION

The authors declare that no funds, grants, or other support were received during the preparation of this manuscript.

## CONFLICT OF INTEREST STATEMENT

Authors declare that they have no conflict of interest.

## CONSENT TO PARTICIPATE

All the co‐authors are willing to participate in this manuscript.

## CONSENT FOR PUBLICATION

All authors are willing for publication of this manuscript.

## Data Availability

The datasets generated used and/ or analyzed during the current study available from the corresponding author on reasonable request.
